# The Varying Coherences of Implied Motion Modulates the Subjective Time Perception

**DOI:** 10.3389/fpsyg.2021.602872

**Published:** 2021-02-25

**Authors:** Feiming Li, Lei Wang, Lei Jia, Jiahao Lu, Youping Wu, Cheng Wang, Jun Wang

**Affiliations:** ^1^Key Laboratory of Intelligent Education Technology and Application of Zhejiang Province, Zhejiang Normal University, Jinhua, China; ^2^College of Education and Human Development, Zhejiang Normal University, Jinhua, China

**Keywords:** implied motion, varying coherence, time perception, temporal bisection task(TBT), point of subjective equality(PSE)

## Abstract

Previous research has demonstrated that duration of implied motion (IM) was dilated, whereas hMT+ activity related to perceptual processes on IM stimuli could be modulated by their motion coherence. Based on these findings, the present study aimed to examine whether subjective time perception of IM stimuli would be influenced by varying coherence levels. A temporal bisection task was used to measure the subjective experience of time, in which photographic stimuli showing a human moving in four directions (left, right, toward, or away from the viewer) were presented as probe stimuli. The varying coherence of these IM stimuli was manipulated by changing the percentage of pictures implying movement in one direction. Participants were required to judge whether the duration of probe stimulus was more similar to the long or short pre-presented standard duration. As predicted, the point of subjective equality was significantly modulated by the varying coherence of the IM stimuli, but not for no-IM stimuli. This finding suggests that coherence level might be a key mediating factor for perceived duration of IM images, and top-down perceptual stream from inferred motion could influence subjective experience of time perception.

## Introduction

Time perception is fundamental for making sense of the dynamic physical world and has been intensively studied (see detailed reviews in Grondin, 2010; Matthews and Meck, 2016). Specifically, how subjective time perception is influenced by the nontemporal stimulus properties fulfilled a large part of previous literature. Many nontemporal stimulus properties have been evidenced to distort subjective time perception, including, but not only, stimulus size (Ono and Kawahara, 2007), brightness (Goldstone et al., 1978; Xuan et al., 2007), loudness of sound (Goldstone et al., 1978), and stimulus weight (Lu et al., 2011). One general finding is that the subjective duration of a given interval increases with the magnitude of the stimulus, which is termed as “magnitude effects” (Matthews and Meck, 2016). For example, bright stimulus was judged as longer than dim stimulus (Goldstone et al., 1978; Xuan et al., 2007), and the subjective time duration of pure tone was reported to increase with the loudness of pure tone (Goldstone et al., 1978). As proposed in the “Processing Principle” (Matthews and Meck, 2016), this magnitude effect suggests that higher intensity enhances perceptual vividity of stimulus, which in turn increases the subjective time duration.

In addition to stimulus intensity, stimulus movement could also enhance perceptual vividity of stimulus and influence the subjective time perception. Visual motion processing is known to modulate subjective time perception (Brown, 1995; Kanai et al., 2006; Yamamoto and Miura, 2012). Previous studies have revealed that geometric moving or flickering stimuli were judged to have a longer duration than static stimuli (Brown, 1995; Kanai et al., 2006). Moreover, processing of both local (e.g., speed) and global motion information (e.g., coherence) could influence time perception (Yamamoto and Miura, 2012, 2016). Consistent reports showed that increased speed of moving stimuli lengthened perceived time duration (Kanai et al., 2006; Kaneko and Murakami, 2009). However, there is a discrepancy regarding the impact of motion coherence on time perception. Through modulating the motion coherence of random dots, Kanai et al. (2006) found no evidence for subjective time duration changing with coherence. In contrast, a study using moving diamond composed of four-line segments showed that perceived duration for coherently moving diamond was either longer or shorter than incoherently moving diamond depending on coherent motion type (circular motion vs. linearly motion), and this perceived duration difference could be attributed to different perceived speed between circular and linearly coherent motion (Yamamoto and Miura, 2016). Furthermore, a transcranial magnetic stimulation (TMS) study showed that disputing the activity of human middle temporal complex (hMT+) via TMS reduced the precision of the temporal judgments (Bueti et al., 2008). These results suggest that motion processing areas (e.g., hMT+) in the dorsal pathway play a key role in time perception (Bueti et al., 2008; Kourtzi et al., 2008).

As a counterpart of real motion (RM), a vivid sense of motion can also be inferred from static pictures of objects in motion such as a cup falling off a shelf, a photograph of an athlete running, etc. This perceptual phenomenon is known as implied motion (IM) and reflects the inferred motion from the top-down perceptual processing (Kourtzi et al., 2008). A set of recent studies has shown that the IM and RM might share similar processing mechanisms. For example, both of them activate the motion-sensitive visual cortex such as the hMT+ of the human extrastriate cortex (Kourtzi and Kanwisher, 2000) and show the similar motion-induced position shift effects (Holmin et al., 2016). Given the processing similarity between RM and IM, subjective time perception has also been reported to be distorted by IM, with longer duration perceived for IM compared to static images (Nather and Bueno, 2011, 2012; Nather et al., 2013). For example, when exposed to photographs of dancer sculptures, participants perceived longer duration compared to exposure to nondancing static sculptures, although both dancing, and nondancing images presented with the same duration (Nather and Bueno, 2011; Nather et al., 2013). In a following study, Yamamoto and Miura (2012) replicated this result by showing that IM evoked by static images of a man running (compared to that of a man standing still) increases the perceived duration of image presentation. Although these studies revealed influences of IM on time perception, research has not yet investigated whether global motion information (e.g., coherence) in IM could modulate the perceptual vividity and distort the subjective time duration. This is partially because most of studies on IM mainly focused on behavioral and neural responses to the single image. Our recent study has demonstrated that varying coherence of IM could modulate brain activations on hMT+ (Jia et al., 2019). In that experiment, four static images showing a human moving in four directions (left, right, toward, or away from the viewer) were presented as probe stimuli, and the varying coherence of these IM stimuli was manipulated by changing the percentage of pictures implying movement in horizontal direction (left or right). Results showed that coherence level-dependent brain activity in motion-sensitive human extrastriate cortex increases with motion coherence (Jia et al., 2019).

The purpose of the present study was to examine whether varying coherence of IM could impact subjective time perception. To this end, we combined the bisection time perception paradigm and our precious IM coherence paradigm. Based on the important role of hMT+ on time perception and our previous findings about increased hMT+ activity along with coherence of IM, we hypothesized that the perceived duration of IM stimuli would be lengthened with increased coherence of IM stimuli. In addition, given that the temporal sensitivity [measured with just noticeable difference (JND) and/or Weber fraction] of IM images is reported to be no different from that of nonimplied images (Nather et al., 2011; Yamamoto and Miura, 2012), we hypothesized that the temporal sensitivity of IM/non-IM images would also be no different across coherence levels.

## Materials and Methods

### Subjects

Twenty-nine healthy subjects (aged 19–26 years; nine men) participated in the experiment. The planned sample size was calculated using statistical power analysis online tool WebPower (Zhang and Yuan, 2018)^1^ based on the experimental design [i.e., two (stimulus type: IM/non-IM) × 4 (coherence level: 0, 50, 75, and 100%)], using the statistical parameters of a repeated-measures analysis of variance (ANOVA) (within factors) as follows: effect size (*F*-test) = 0.25, α err probability = 0.05 (two-tailed), power (i.e., 1-β_err probability) = 0.8, number of groups = 1, number of measurements = 8, correlations among repeated measures = 0.5, and nonsphericity correlation ε = 1. The results returned a planning sample size of 29. Accordingly, we recruited 29 participants, and two of them were excluded because of unsuccessfully fitting the psychometric curve (*R*^2^ < 0.8). In addition, for all the left data with *R*^2^ > 0.8, a *χ*^2^ test was used to test the goodness of fit. Results showed that the data for each participant fitted from a distribution of a logistic function (*χ*^2^s ≤ 8.67, *p* ≥ 0.19). All participants were right-handed and had normal or corrected-to-normal vision. The research ethics committee of Zhejiang Normal University approved the study. Participants were provided written informed consent (in accordance with the Declaration of Helsinki) prior to testing and were paid ¥20 for their participation.

### Stimuli and Procedures

**FIGURE 1:**
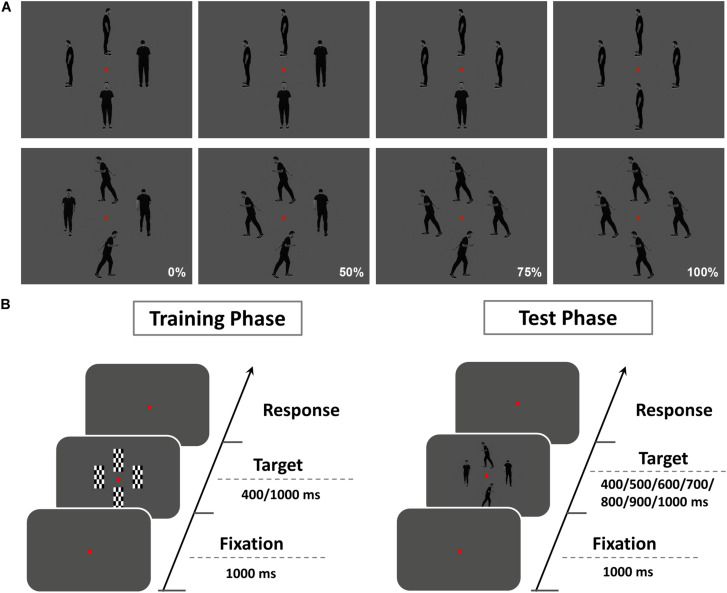
**(A)** The illustration for stimuli used in the experiment. Two types of gray-scale pictures were used as test stimuli: implied motion (IM; top row), non–implied motion (non-IM; bottom row). IM stimuli consisted of three sets of four images of a single person surrounding a central fixation point at a distance of two degrees. In each image, the human agent was running left, right, toward, or away from the viewer. The non-IM stimuli also consisted of three similar sets of images, but in these images, the human agent was shown in a standing position, leaning left, right, toward, or away from the viewer. Coherence was operationalized as the percentage of images facing toward or moving in a single horizontal direction and composed of four levels (0, 50, 75, and 100%). **(B)** Schematic diagram showing the procedure of the present study. The whole experiment was divided into two sessions: an initial training phase and a follow-up test phase.

Stimuli were presented on a 24-inch monitor at a viewing distance of 60 cm. Similar to our previous study (Jia et al., 2019), two types of gray-scale pictures were used as test stimuli: IM and non-IM (Figure 1A). IM stimuli consisted of three sets of four images of a single person surrounding a central fixation point at a distance of two degrees. In each image, the human agent was running left, right, toward, or away from the viewer. The non-IM stimuli also consisted of three similar sets of images, but in these images, the human agent was shown in a standing position, leaning left, right, toward, or away from the viewer. Coherence was operationalized as the percentage of images facing toward or moving in a single horizontal direction and composed of four levels (0, 50, 75, and 100%). To control the possible speed information differences conveyed by different coherence level of IM stimuli, IM stimuli at each coherent level were rated by a group of 24 independent naive observers following a similar procedure in previous studies (Williams and Wright, 2009; Lu et al., 2016). It was confirmed that there were no significant perceived speed differences for IM stimuli at different coherent levels [*F*_(3,69)_ = 0.58, *p* = 0.63, η_*p*_^2^ = 0.03].

Here we used the temporal bisection task (TBT) (Yamamoto and Miura, 2012) to examine the perceived duration of IM and non-IM images at various coherence levels, respectively. This task included a training phase followed by a test phase (Figure 1B). In the training phase, participants were presented with a set of four black–white checkerboard images (training stimulus). Each image surrounded a central fixation point at a distance of two degrees. Training stimuli were presented with two standard durations (400 and 1,000 ms) after a 1-s fixation display, and participants were instructed to categorize the training stimulus as “long” or “short.” Each standard duration was repeated five times, and the order of presentation of the two standard durations was randomized. Participants passed the training phase after completing 10 consecutive correct responses. In the test phase, participants were instructed to fixate on the red dot at the center of test stimulus (IM or non-IM stimulus) and asked to judge whether the duration of test stimulus was more similar to the long or short standard duration. Test stimulus was presented with seven probe durations (400, 500, 600, 700, 800, 900, and 1,000 ms) after a 1-s fixation display. The response keys were counterbalanced across observers. Each participant completed 840 trials, which consisted of 15 trials for each probe duration and for each trial type [8 trial types: 2 stimulus type (IM, non-IM) × 4 coherence levels (0, 50, 75, and 100%)]. The whole test phase included five blocks, each of 168 trials. The trial order was randomized across blocks and across participants.

### Data Analysis

The proportions of “long” responses were calculated for the seven probe durations and fitted by a logistic function (Treutwein and Strasburger, 1999) for each stimulus type and for each coherence level, respectively. The point of subjective equality (PSE) was then calculated based on the 50% point in the obtained logistic curve. To further examine the sensitivity of the TBT, we calculated the JND of the temporal bisection using half of the difference in duration between the 25 and 75% point (Shi et al., 2008; Vroomen and Keetels, 2010), as well as the Weber fraction dividing JND by the PSE.

## Results

### PSE

First, we conducted a two (stimulus type: IM/non-IM) × 4 (coherence level: 0, 50, 75, and 100%) repeated-measures ANOVA on PSE. The ANOVA results revealed a significant main effect of the stimulus type [*F*_(__1,26)_ = 11.71, *p* = 0.002, η_*p*_^2^ = 0.31], but the main effect of coherence level was not significant [*F*_(3,78)_ = 0.63, *p* = 0.58, η_*p*_^2^ = 0.02]. *Post hoc* tests showed that PSE for IM stimulus (mean = 638.86 ms, SE = 11.76 ms) was significantly smaller than non-IM stimulus (mean = 651.84 ms, SE = 11.56 ms).

**FIGURE 2:**
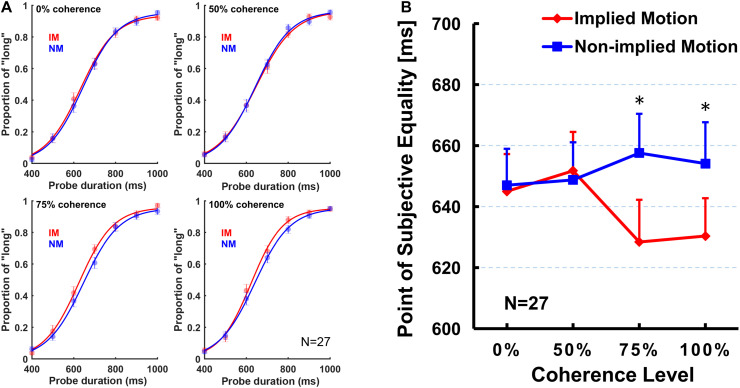
The obtained logistic curves of the “long” responses **(A)** and mean point of subjective equality (PSE; **B**) for each stimulus type and for each coherence level. Error bars represent standard errors of the mean across subjects. Sample size *n* = 27.

In addition, there was a significant stimulus type × coherence level interaction [*F*_(__3,78)_ = 4.39, *p* = 0.015, η_*p*_^2^ = 0.15]. The simple effect analysis showed that the PSE for IM stimulus was significantly smaller than non-IM stimulus, respectively, at 75% [*F*_(__1,26)_ = 9.09, *p* = 0.006, η_*p*_^2^ = 0.26] and 100% coherence levels [*F*_(__1,26)_ = 12.62, *p* = 0.001, η_*p*_^2^ = 0.33], but no difference between stimulus types at 0% [*F*_(__1,26)_ = 0.15, *p* = 0.70, η_*p*_^2^ = 0.01] or 50% coherence levels [*F*_(__1,26)_ = 0.14, *p* = 0.71, η_*p*_^2^ = 0.01; Figure 2]. Furthermore, separate repeated-measures ANOVAs were conducted on PSE for IM and non-IM stimuli. The PSE for IM stimulus showed a significant main effect of coherence level [*F*_(__3,78)_ = 3.67, *p* = 0.025, η_*p*_^2^ = 0.12]. Multiple comparisons among coherence levels were analyzed by least significant difference *post hoc* test. Results showed that PSE at 100% coherence level was significantly smaller than those at 0% (*p* = 0.046) and 25% (*p* = 0.013), and PSE at 75% coherence level was significantly smaller than those at 25% (*p* = 0.040) and marginally significantly smaller than those at 0% (*p* = 0.071). No other pairwise differences between coherence levels were found. In contrast, the main effect of coherence level of the PSE for the non-IM stimulus was not significant [*F*_(__3,78)_ = 0.65, *p* = 0.57, η_*p*_^2^ = 0.03].

### JND

**FIGURE 3:**
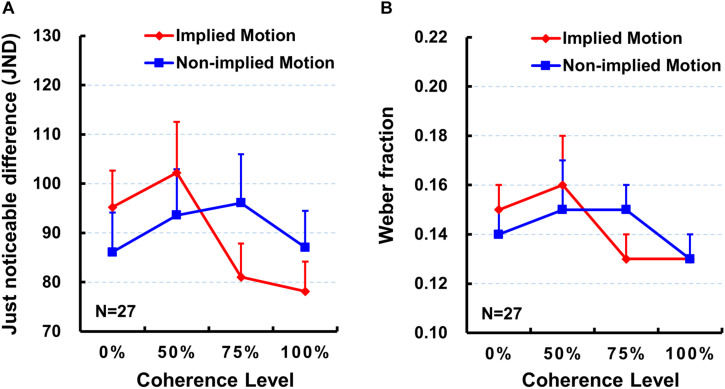
Mean just noticeable difference (JND; **A**) and mean Weber fraction **(B)** for each stimulus type and for each coherence level. Error bars represent standard errors of the mean across subjects. Sample size *n* = 27.

Next, we did the same repeated-measure ANOVA on the JND. However, neither main effects [*F*’s < 2.20, *p* > 0.10, η_*p*_^2^ < 0.08] nor interaction effects [*F*_(__3,78)_ = 2.40, *p* = 0.091, η_*p*_^2^ = 0.08] were significant (Table 1 and Figure 3A).

**TABLE 1:** The descriptive statistics results of PSE, JND, and Weber fraction (mean ± SE).

	Stimulus type	Coherence level			
		0%	50%	75%	100%
PSE	IM	644.96 ± 12.26	651.74 ± 12.76	628.41 ± 13.81	630.33 ± 12.43
	Non-IM	647.00 ± 11.91	648.74 ± 12.30	657.56 ± 12.85	654.07 ± 13.58
JND	IM	95.22 ± 7.46	102.20 ± 10.33	81.04 ± 6.82	78.15 ± 6.06
	Non-IM	86.11 ± 8.03	93.61 ± 9.32	96.11 ± 9.89	87.06 ± 7.40
Weber fraction	IM	0.15 ± 0.01	0.16 ± 0.02	0.13 ± 0.01	0.13 ± 0.01
	Non-IM	0.14 ± 0.01	0.15 ± 0.02	0.15 ± 0.01	0.13 ± 0.01

### Weber Fraction

Finally, we did the same repeated-measure ANOVA on the Weber fraction. As with the JND, neither main effects [*F*’s < 2.31, *p* > 0.09, η_*p*_^2^ < 0.08] nor interaction effects [*F*_(__3,78)_ = 1.62, *p* = 0.20, η_*p*_^2^ = 0.06] were significant (also see Table 1 and Figure 3B).

## Discussion

The present study was designed to examine whether varying coherence of IM influences perceived presentation duration. In addition to comparing subjective perceived duration of the depictions of movement (IM) and nonmovement (non-IM) of the same human characters as previous studies (e.g., Nather and Bueno, 2011, 2012; Yamamoto and Miura, 2012, 2016; Nather et al., 2013), we were particularly interested at comparisons between IM and non-IM at varying coherence defined by the percentage of images facing toward or moving in a single horizontal direction. Results indicated that IM images were perceived to be presented for longer than non-IM images at a higher coherence level (75 and 100%; Figure 2), but not at a lower coherence level (0 and 50%). Moreover, perceived duration of IM images exhibited significantly increasing trend from low to high coherence, while perceived duration of non-IM images remained stable (Figure 2). Finally, the sensitivity of the temporal judgment (JND and Weber fraction) did not show any significant difference between IM and non-IM or across varying coherence levels (Figure 3). These findings suggest that varying coherences of IM image increase the perceived duration of the stimulus.

Compared to previous studies, there are two distinct findings observed in the present study. First, unlike previous studies showing perceived longer duration of single IM image than non-IM image (Nather and Bueno, 2011, 2012; Yamamoto and Miura, 2012), our results indicated that perceived duration of IM images was only longer than non-IM images at a high coherence level (75 and 100%). This discrepancy may be attributed to a difference in stimulus configurations. Previous studies used a single IM image, whereas the current study used a stimulus composed of four different oriented images. Given the fact that IM image at either orientation causes the time dilation effect (Yamamoto and Miura, 2012), time dilation effect could be canceled out when different oriented IM images were presented simultaneously (e.g., stimulus configuration at 0 and 50% coherence levels). Considering the role of motion processing areas (e.g., hMT+) in time perception (Kaneko and Murakami, 2009), it is reasonable to conclude that hMT+ activity influenced by coherence is the key to determine the perceived duration, and the relatively smaller hMT+ activity at a low coherence level due to different oriented IM images leads to canceled-out time dilation effect observed in the current study. Therefore, the results of the current study and our previous study supported the “intrinsic models” of timing, which proposed that estimation of time duration depending on the magnitude of neural activity evolved during the passage of time (Ivry and Schlerf, 2008). In addition, our results found a significantly increasing trend from low to high coherence on the perceived duration of IM images but not on that of non-IM images. According to the “Processing Principle” proposed in a previous study (Matthews and Meck, 2016), this finding suggested that increased coherence levels may specifically enhance the perceptual vividity of IM images, which lengthened the subjective time duration accordingly. Second, by using RM stimuli, there is a discrepancy regarding the impact of motion coherence on time perception (Kanai et al., 2006; Yamamoto and Miura, 2016). Some found no evidence for subjective time duration changing with coherence (Kanai et al., 2006), whereas some showed that perceived duration for coherently moving stimuli was either longer or shorter than incoherently moving stimuli, depending on different perceived speed over coherent motion type (circular motion vs. linearly motion; Yamamoto and Miura, 2016). On the contrary, our results indicated that perceived duration of IM stimuli was lengthened with increased coherence, which was independent of perceived speed as no subjective speed difference was reported among different coherence levels. This finding indicated that IM stimuli configuration used in the present study could relatively easily separate speed and coherence features. By contrast, RM usually generates specific spatiotemporal changes (Brown, 1995; Kanai et al., 2006; Kaneko and Murakami, 2009), and it is hardly separating these two features in RM stimuli (Yamamoto and Miura, 2016).

It is worth mentioning that IM images were perceived to be presented for longer than non-IM images at a higher coherence level (75 and 100%), but not at a lower coherence level (0 and 50%), whereas our previous study (Jia et al., 2019) showed neural activity in hMT+ exhibited significant difference at coherence levels of 50, 75, and 100%. This discrepancy suggests that low-level visual features such as coherence (i.e., global orientation) may drive the motion detection system, which in turn impacts the time perception. Moreover, this discrepancy suggests that motion selectivity of the extrastriate visual cortex (e.g., hMT+) is grounded in perceptual integration of low-level visual features (e.g., orientation). Therefore, current results could be attributed to a perceptual integration of the top-down corticocortical influences and sensitivity of the low-level visual features (e.g., orientation). Alternative possible reason for this discrepancy is that the neural response measured in our previous study (Jia et al., 2019) might be more sensitive than the behavioral responses in the current study when differentiating IM from non-IM images. Another concern is that current findings might be attributed to the function of memory as participants were required to learn the standard durations and compared them with probe stimuli in the following bisection task. However, previous literature indicated that perceptual errors instead of errors in reference memory dominated the psychometric function in TBT, and the scalar variability was independent of the structure of the bisection task (Allan and Gerhardt, 2001; Rodríguez-Gironés and Kacelnik, 2001; Wearden and Bray, 2001). For example, Allan and his colleagues compared the scalar variability among three structures of the bisection task: no-referent condition, in which the referent pairs (short/long) were presented at the beginning of experiment (same as ours); fixed-referent condition, in which the referent pair with fixed time length was presented at the beginning of each probe trial; and roving-referent condition, in which the referent pair with varied time length was presented at the beginning of each probe trial. The results showed that there was no difference on the scalar variability across three conditions, and probe was compared with the criterion (i.e., bisection point) instead of referents even when referents were available on each trial. Based on these evidences, we would be ensured that memory would not be a confounding factor in the present study. However, further work employing the fixed-referent temporal bisection paradigm needs to be done to confirm this empirically.

Overall, although our study did not provide a direct evidence as to the possible neural substrates of the effects varying coherence of IM on time perception, the observation that the perceived duration of IM lengthened with increased coherence suggests that coherence level might be a key mediating factor for perceived duration of IM images.

## Data Availability Statement

The raw data supporting the conclusions of this article will be made available by the authors, without undue reservation.

## Ethics Statement

The studies involving human participants were reviewed and approved by the Research Ethics Committee of Zhejiang Normal University (ZJNU). The patients/participants provided their written informed consent to participate in this study.

## Author Contributions

FL: experiment design, data analysis, and draft writing. LW: data collection, experiment design, and draft writing. LJ: experiment design and draft writing. JL: data analysis. YW: data analysis. CW: data analysis. JW: research conception, experiment design, and draft writing. All authors contributed to the article and approved the submitted version.

## Conflict of Interest

The authors declare that the research was conducted in the absence of any commercial or financial relationships that could be construed as a potential conflict of interest.

## Footnotes

1 https://webpower.psychstat.org/wiki/
